# 3M^™^ Petrifilm^™^ Rapid Aerobic Count (RAC) Plate Method for the Enumeration of Aerobic Bacteria on Selected Surfaces: AOAC *Official Method*^SM^ 2015.13

**DOI:** 10.1093/jaoacint/qsac122

**Published:** 2022-10-17

**Authors:** Cari K Lingle, April J Schumacher, Micki L Rosauer, Karen M Silbernagel, Andrew Deterding

**Affiliations:** 3M Company, Food Safety Department, St. Paul, MN 55144-1000,USA; 3M Company, Food Safety Department, St. Paul, MN 55144-1000,USA; 3M Company, Food Safety Department, St. Paul, MN 55144-1000,USA; 3M Company, Food Safety Department, St. Paul, MN 55144-1000,USA; Q Laboratories, Cincinnati, OH 45204, USA

## Abstract

**Background:**

The 3M^™^ Petrifilm^™^ Rapid Aerobic Count (RAC) Plate is a sample-ready culture medium system designed to facilitate colony enumeration in 24 h (48 h for dry powders) for selected food and environmental surfaces.

**Objective:**

The objective of this study is to evaluate the 3M Petrifilm RAC Plate in a matrix extension study for the enumeration of aerobic bacteria on stainless steel, sealed concrete, and rubber surfaces.

**Method:**

The 3M Petrifilm RAC Plate was compared to the U.S. Food and Drug Administration *Bacteriological Analytical Manual*, Ch. 3 (January 2001): *Aerobic Plate Count* in a paired matrix study for enumeration of aerobic bacteria on stainless steel, sealed concrete, and rubber environmental surfaces.

**Results:**

The 3M Petrifilm RAC Plate showed no statistically significant differences when compared to the reference method for enumeration of aerobic bacteria from stainless steel, sealed concrete, and rubber environmental surfaces. There were no significant statistical differences between the 3M Petrifilm RAC Plate and reference method results for the three matrixes evaluated.

**Conclusions:**

The 3M Petrifilm RAC Plate is an effective plating method for aerobic plate count when analyzing stainless steel, sealed concrete, and rubber surfaces.

**Highlights:**

The 3M Petrifilm RAC Plate is robust, quick, and simple to perform, providing enumerable colonies in 22 to 26 h after incubation when compared to the 48 h of the reference method. The small size of the Petrifilm Plate allows for less space to be occupied by plates in the incubators. The visual biochemical and enzymatic indicators make enumeration of colonies a simple task by presenting colonies in either a blue or red color.

Aerobic plate counts provide food manufacturers with an indication of the overall titer of aerobic bacteria in the product being tested. Any surfaces used for food production may also be assessed for a level of aerobic bacteria to provide a general indication of the cleanliness of food production areas and equipment. Traditional methods using agar for the determination of an aerobic count from food require 48 h of incubation time, whereas the 3M Petrifilm Rapid Aerobic Count (RAC) Plate was developed to provide enumeration of total aerobic count for the food and beverage industries in as little as 24 h.

The 3M Petrifilm RAC Plate is a culture medium that is sample-ready out of the package. The 3M Petrifilm RAC Plate includes nutrients required for the enumeration of aerobic bacteria from samples. The plate also includes a cold-water-soluble gelling agent and a dual-sensing indicator technology. The combination of the nutrients, gelling agent, and dual-sensing indicator allow for colony enumeration in 24 h (48 h for dry powders).

After lifting the top film, the sample is first added to the center of the 3M Petrifilm RAC Plate, and then the top film is released. A flat plastic sample spreader is placed on the closed, inoculated plate, and then the sample is spread over the plate using light pressure on the spreader. The plate is allowed to sit for 1 min to allow the gelling agent to solidify. The nutrients contained in the plate allow for aerobic bacteria growth when plates are incubated at the correct temperature. After 24 h of incubation, plates can be analyzed. The dual-sensing indicator technology makes aerobic bacterial growth appear as blue or red colonies on the 3M Petrifilm RAC Plate. The plates can then be easily read using a standard colony counter or magnifying source (1). 3M Petrifilm RAC Plates do not differentiate any one microorganism strain from another.

The 3M Petrifilm RAC Plate was previously shown to be comparable to the U.S. Food and Drug Administration *Bacteriological Analytical Manual* (BAM) Chapter 3: *Aerobic Plate Count* (2) and Standard Methods for the Examination of Dairy Products (SMEDP) Chapter 6: *Microbiological Count Methods* (3) in approximately 24 h less than the standard methods. The 3M Petrifilm RAC Plate was validated using representative samples of the following matrixes: raw ground beef, raw ground pork, chicken carcass rinsate, raw turkey sausage, fresh swai, fresh tuna, fresh tiger shrimp, easy-peel shrimp, cherry tomato wash, frozen blueberries, Mediterranean apricots, creamy salad dressing, fresh pasta, vanilla ice cream, dry milk powder, and pasteurized skim milk (4–6).

This matrix extension study compares the 3M Petrifilm RAC Plate method to BAM Chapter 3, which uses Plate Count Agar (PCA) for the enumeration of aerobic bacteria on stainless steel, sealed concrete, and rubber.

## Matrix Extension Validation Study

This study was conducted under the AOAC *Performance Tested Method*^SM^ program and the *AOAC INTERNATIONAL Methods Committee Guidelines for Validation of Microbiological Methods for Food and Environmental Surfaces* (7). The independent laboratory study was conducted by Q Laboratories (Cincinnati, OH), comparing the 3M Petrifilm RAC Plate method to the BAM Ch. 3 reference method using a paired study design for stainless steel (18 GA, 300 series, brushed finish), sealed concrete, and rubber (ethylene propylene diene monomer).

### Matrix Study

The organisms used in the matrix study were obtained from the American Type Culture Collection (ATCC, Manassas, VA): *Listeria innocua* ATCC 33090 (source: cow brain), *Klebsiella* (formerly *Enterobacter*) *aerogenes* ATCC 35029 (origin not available), and *Klebsiella oxytoca* ATCC 43165 (source: clinical isolate).

Stainless steel, sealed concrete, and rubber were provided by the independent laboratory and chemically disinfected with an EPA-regulated quaternary ammonium solution. The solution was added to the surface and remained wet for 10 min for proper disinfection according to the product technical data sheet. After 10 min the excess liquid was removed, and the surface was allowed to dry. After disinfection, the environmental surfaces were artificially contaminated as follows: *L. innocua* (ATCC 33090) was used to inoculate stainless steel, *K. aerogenes* (ATCC 35029) was used to inoculate rubber, and *K. oxytoca* (ATCC 43165) was used to inoculate sealed concrete.

The study consisted of evaluating a total of 20 paired 100 cm^2^ samples of stainless steel, 20 paired 100 cm^2^ samples of sealed concrete, and 20 paired 100 cm^2^ samples of rubber. Within each sample set of 20, there were five uninoculated portions (0 colony-forming units/100 cm^2^), five portions inoculated at 1–100 colony-forming units/100 cm^2^, five portions inoculated at 100–1000 colony-forming units/100 cm^2^, and five portions inoculated at 1000–10 000 colony-forming units/100 cm^2^. All test portions were surfaces sampled using the 3M Swab Sampler with 10 mL of Letheen broth and analyzed with the 3M Petrifilm RAC Plates and BAM Ch. 3 methods.

All isolates used to inoculate the stainless steel, sealed concrete, and rubber were prepared by propagating from a stock culture stored at −70°C to trypticase soy agar with 5% sheep blood (SBA) and incubating for 24 ± 2 h at 35 ± 1°C. A single colony was then transferred from SBA to brain heart infusion (BHI) broth and incubated at 35 ± 1°C for 24 ± 2 h. After incubation, the culture was diluted to a target level using BHI broth as the diluent and added to the matrix at an appropriate amount, accounting for dieoff, where five portions were inoculated with sterile BHI broth (0 colony-forming units/100 cm^2^), five portions were inoculated at 1–100 colony-forming units/100 cm^2^, five portions were inoculated at 100–1000 colony-forming units/100 cm^2^, and five portions were inoculated at 1000–10 000 colony-forming units/100 cm^2^. The test area was inoculated with 0.25 mL to allow for even distribution of the inoculum over the entire test surface. The inoculated environmental surfaces were allowed to dry for 16–24 h at room temperature (18–25°C). The surface of each matrix was visibly dry at time of sample collection. All test areas were sampled using horizontal and vertical strokes using firm pressure while rotating the head of the swab to ensure the entire area was sampled. The swabs were placed back into their original containers. For this study, swabs were held at room temperature (18–25°C) for 2 h before further processing. Swabs were thoroughly homogenized by vortex prior to performing dilutions and plating.

### Candidate Method

For the 3M Petrifilm RAC Plate candidate method, the paired 100 cm^2^ test portions of stainless steel, sealed concrete, and rubber were sampled using the 3M Swab Sampler with 10 mL Letheen broth. All test areas were sampled and the swabs held before further processing as described previously.

A 1:10 serial dilution was conducted by transferring 1 mL of product into 9 mL of Butterfield’s Phosphate Buffer. The appropriate serial dilutions were conducted from each swab sample to achieve counts within the countable range. Each sample dilution was homogenized thoroughly, and 1 mL of each sample pipetted into separate, duplicate 3M Petrifilm RAC Plates by lifting the plates top film and pipetting the sample onto the center of the bottom film. The top film was rolled down gently, and the sample was spread using a spreader. The 3M Petrifilm RAC Plates were then left undisturbed for 1 min to allow the gel in the plate to form.

The plates were placed in an incubator set to 35 ± 1°C for 24 ± 2 h in stacks of no more than 40. After incubation, the plates were removed and colonies enumerated for each dilution and matrix.

### Reference Method

For the BAM Ch. 3 reference method, the dilutions from each surface preparation for the 3M Petrifilm RAC Plate method were also used for the BAM Ch. 3 reference method. Each sample dilution tube was vortexed thoroughly, and 1 mL was pipetted into separate, duplicate Petri dishes. PCA (12–15 mL at 45 ± 1°C) was added to each plate within 15 min of original dilution. Samples and agar were mixed by rotating and moving plates back and forth on a flat surface. The agar was then allowed to solidify.

Solidified plates were then inverted and placed in an incubator set to 35 ± 1°C for 48 ± 2 h. After 48 ± 2 h, plates were removed from incubator. The number of colonies per dilution and per environmental surface type were enumerated and recorded.

**AOAC *Official Method*^SM^ 2015.13**

**Enumeration of Aerobic Bacteria in Food and**

**Selected Surfaces 3M Petrifilm Rapid**

** Aerobic Count Plate**

**First Action 2015**

**Final Action 2018**

[Applicable to the enumeration of aerobic bacteria from raw ground beef, raw ground pork, raw ground turkey, chicken carcass rinsate, fresh swai, fresh tuna, fresh tiger shrimp, raw easy-peel shrimp, cherry tomato wash, frozen blueberries, Mediterranean apricots, creamy salad dressing, fresh pasta, vanilla ice cream, instant nonfat dry milk (NFDM), pasteurized skim milk, stainless steel, sealed concrete, and rubber.]

*Caution*: After use, the diluents and 3M Petrifilm RAC Plates may contain microorganisms that may be a potential biohazard. When testing is complete, follow current industry standards for the disposal of contaminated waste. Consult the Material Safety Data Sheet for additional information and local regulations for disposal.

To reduce the risks associated with bacterial infection and workplace contamination, perform 3M Petrifilm RAC Plate testing in a properly equipped laboratory under the control of a skilled microbiologist. The user must train personnel in current proper testing techniques—for example, good laboratory practices, ISO 17025, or ISO 7218.

*See* Tables **2015.13A** and B for results of the interlaboratory study supporting acceptance of the method.

**Table 2015.13A.** Interlaboratory study results of 3M Petrifilm RAC Plate versus FDA BAM Chapter 3 method for raw easy-peel shrimp

Matrix	3M Petrifilm RAC Plate					FDA BAM Chapter 3					Difference of means	Difference of means 95% LCL, UCL^d^^,^^e^	Reverse-transformed difference of mean, colony-forming units/g	Reverse-transformed difference of means LCL, UCL
	Lot	*N* ^a^	Mean log_10_ cfu/g	$s_{r}^{}$ ^b^	$s_{R}^{}$ ^c^	Lot	*N*	Mean log_10_ cfu/g	s_r_	s_R_				
Raw easy-peel shrimp 32°C	Low	16	2.96	0.132	0.280	Low	16	3.02	0.218	0.356	0.06	–0.11, 0.24	139.47	0.77, 1.72
	Medium	16	4.29	0.202	0.215	Medium	16	4.23	0.095	0.298	–0.06	–0.18, 0.06	–2424.10	0.67, 1.15
	High	16	5.56	0.110	0.248	High	16	5.76	0.097	0.214	0.20	–0.01, 0.42	214352.79	0.97, 2.61
Raw easy-peel shrimp 35°C	Low	16	2.80	0.121	0.335	Low	16	3.02	0.218	0.356	0.22	–0.03, 0.48	422.68	0.92, 3.03
	Medium	16	4.22	0.172	0.273	Medium	16	4.23	0.095	0.298	0.01	–0.08, 0.11	539.37	0.83, 1.28
	High	16	5.67	0.141	0.174	High	16	5.76	0.097	0.214	0.09	–0.09, 0.26	105217.30	0.82, 1.83

a *N* = Number of laboratories that reported complete results.

b s_r_ = Repeatability.

c s_R_ = Reproducibility.

d LCL, UCL = 95% lower and upper confidence limits, respectively.

e A 95% confidence interval that contains the point 0 indicates no statistically significant difference between methods.

**Table 2015.13B.** Interlaboratory study results of 3M Petrifilm RAC Plate versus SMEDP Chapter 6 method for pasteurized skim milk and instant NFDM

Matrix	3M Petrifilm RAC Plate					SMEDP Chapter 6					Difference of means	Difference of means 95% LCL, UCL^d^^,^^e^	Reverse-transformed difference of mean, colony-forming units/g	Reverse-transformed difference of means LCL, UCL
	Lot	*N* ^a^	Mean log_10_ cfu/g	$s_{r}^{}$ ^b^	$s_{R}^{}$ ^c^	Lot	*N*	Mean log_10_ cfu/g	s_r_	s_R_				
Pasteurized skim milk	Low	13	2.51	0.131	0.310	Low	13	2.47	0.123	0.301	–0.04	–0.08, 0.01	24.56	0.83, 1.03
	Medium	13	3.53	0.180	0.242	Medium	13	3.48	0.119	0.264	–0.05	–0.13, 0.03	346.20	0.75, 1.08
	High	13	4.63	0.136	0.232	High	13	4.58	0.116	0.196	–0.05	–0.11, 0.01	4936.41	0.78, 1.00
Instant NFDM	Low	15	2.42	0.096	0.126	Low	15	2.34	0.129	0.179	–0.08	–0.16, 0.01	42.05	0.69, 1.02
	Medium	15	3.04	0.059	0.148	Medium	15	2.98	0.104	0.195	–0.06	–0.14, 0.01	153.18	0.73, 1.02
	High	15	4.26	0.174	0.190	High	15	4.19	0.185	0.197	–0.07	–0.14, 0.01	2806.94	0.71, 1.00

a *N* = Number of laboratories that reported complete results.

b s_r_ = Repeatability.

c s_R_ = Reproducibility.

d LCL, UCL = 95% lower and upper confidence limits, respectively.

e A 95% confidence interval that contains the point 0 indicates no statistically significant difference between methods.

### A. Principle

The 3M Petrifilm Rapid Aerobic Count (RAC) Plate is a sample-ready culture medium system that contains nutrients, a cold-water-soluble gelling agent, and an indicator system that facilitates aerobic bacterial enumeration. 3M Petrifilm RAC Plates are used for the enumeration of aerobic bacteria in as little as 24 h for most food matrixes. 3M Food Safety is certified to ISO (International Organization for Standardization) 9001 for design and manufacturing.

### B. Apparatus and Reagents

- *3M Petrifilm RAC Plate*.—Available from 3M Food Safety, St. Paul, MN, USA; Cat. No. 6478/6479.
- *Sterile diluent.—*Butterfield’s Phosphate-Buffered Diluent.
- *Pipets.—*Capable of pipetting 1000 μL or a serological pipet.
- *Sterile pipet tips.—*Capable of 1000 μL.
- *Stomacher.—*Seward or equivalent.
- *Filter stomacher bags.—*Seward or equivalent.
- *3M Petrifilm flat spreader.—*Cat. No. 6425.
- *3M Swab Sampler with 10 mL Letheen broth.—*Cat. No. RS96010LET or equivalent.
- *Incubators.—*Capable of maintaining 32 ± 1°C and 35 ± 1°C and having a solid front to maintain a dark interior.
- *Refrigerator or freezer.—*Capable of maintaining temperature between –20 and 8°C for storing unopened 3M Petrifilm RAC Plates.
- *Freezer.—*Capable of maintaining temperature at less than –15°C for storing 3M Petrifilm RAC pouches after incubation.
- *Standard colony counter or illuminated magnifier.*

### C. General Instructions

- *Storage conditions.—*Store the 3M Petrifilm RAC Plates at –20 to 8°C. After opening the 3M Petrifilm RAC Plate pouches, seal the pouch and store at ambient temperature, <60% relative humidity. Post-incubation 3M Petrifilm RAC Plates can be stored at less than –15°C for up to 1 week.
- *Spreader.—*Place the 3M Petrifilm Flat Spreader on the center of the plate when preparing the sample aliquot to prevent trapping air bubbles.
- Follow all instructions carefully. Failure to do so may lead to inaccurate results.

### D. Sample Preparation

- Aseptically prepare a 1:10 dilution of each test portion.
  1. *Dairy products.—*Pipet 11 mL or weigh 11 g of the sample into 99 mL sterile Butterfield’s phosphate-buffered diluent.
  2. *All other foods.—*Weigh a 50 g test portion into a sterile stomacher bag and dilute with 450 mL Butterfield’s phosphate-buffered diluent; blend or homogenize per standard.
  3. *Environmental surfaces.—*Mix or shake swab in Letheen vigorously.
- Prepare 10-fold serial dilutions in Butterfield’s phosphate-buffered diluent. Environmental surface samples may be plated directly as needed.
- Place the 3M Petrifilm RAC Plates on a flat, level surface for each dilution to be tested.
- Lift the film. With the pipet perpendicular, dispense 1 mL of each dilution onto the center of the bottom film of the plate.
- Roll the film down onto the sample.
- Place the 3M Petrifilm Flat Spreader on the center of the plate. Press gently on the center of the spreader to distribute the sample evenly. Spread the inoculum over the entire 3M Petrifilm RAC Plate growth area before the gel is formed. Do not slide the spreader across the film.
- Remove the spreader and leave the plate undisturbed for at least 1 min to permit the gel to form.
- Incubate the 3M Petrifilm RAC Plate at either 32 ± 1°C (seafood and dairy products) or 35 ± 1°C (all other foods and environmental surfaces) in a horizontal position with the clear side up in stacks of no more than 20 (for dairy products), or 40 for all other foods. Enumerate plates after 24 ± 2 h of incubation (or 48 ± 3 h in the case of dairy powders, including whey powder). 3M Petrifilm RAC Plates can be counted using a standard colony counter with the use of a backlight or an illuminated magnifier to assist with the estimated enumeration.
- Enumerate all colonies regardless of size, color, or intensity.
- The circular growth area is approximately 30 cm^2^. Plates containing >300 colonies can be either estimated or recorded as too numerous to count (TNTC). Estimation can be done only by counting the number of colonies in one or more representative squares and determining the average number per square. The average number can be multiplied by 30 to determine the estimated count per plate. If a more accurate count is required, the sample may need to be retested at higher dilutions.
- Report final results as colony-forming units per gram or milliliter (colony-forming units/g or colony-forming units/mL).
*Note*: If there are two dilutions within the countable range, use the following calculation to determine the final count:
  N=ΣC/(1.1×d)
  where *N* = number of colonies per milliliter or per gram of product; ΣC = sum of all colonies on both plates; and *d* = dilution from which first counts were obtained.
- Food samples may occasionally show interference on the 3M Petrifilm RAC Plates—for example:
  1. Uniform blue background color (often seen from the organisms used in cultured products). These should not be counted as TNTC.
  2. Intense pinpoint blue specs (often seen with spices or granulated products).
- When necessary, colonies may be isolated for further identification test using standard procedures. Lift the top film and pick the colony from the gel.

## Results

**Figure 1.**
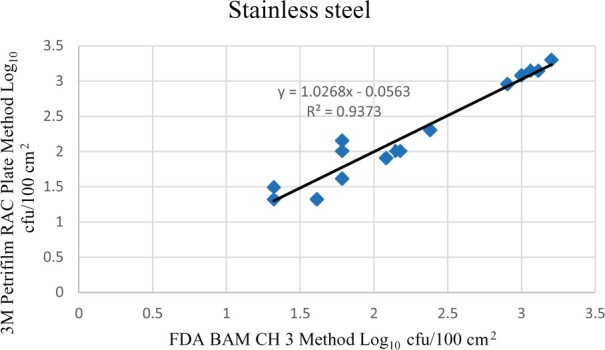
Method comparison results of 3M Petrifilm RAC Plate versus FDA BAM Ch. 3 for stainless steel.

**Figure 2.**
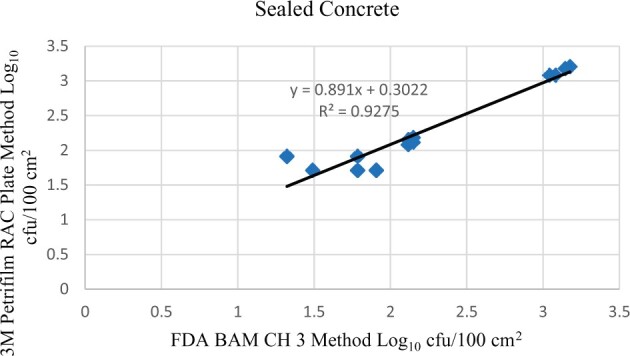
Method comparison results of 3M Petrifilm RAC Plate versus FDA BAM Ch. 3 for sealed concrete.

**Figure 3.**
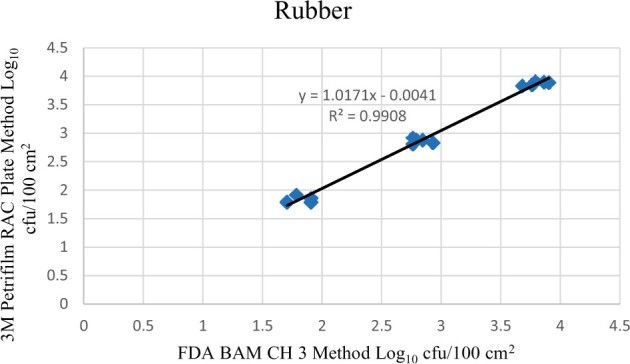
Method comparison results of 3M Petrifilm RAC Plate versus FDA BAM Ch. 3 for rubber.

Statistical analysis was conducted for each contamination level for each matrix. Logarithmic transformation of the counts (colony-forming units/100 cm^2^) was performed, and the difference of means, with 90 and 95% confidence intervals, between the candidate method and the reference method was determined for each matrix and each contamination level. The differences of means and confidence intervals were calculated using the paired method analysis for Micro Testing version 1.2 (Least Cost Formulations, Ltd., Virginia Beach, VA). The 95% confidence interval of the bias between the two methods fell between −0.5 and 0.5 log_10_ for each concentration, indicating equivalence between the two methods, and it exceeded the minimum acceptance criteria recommended in the draft AOAC *Standard Method Performance Requirements* (90% confidence interval of the bias between the two methods is between −0.5 and 0.5 log_10_) (8). The repeatability, calculated as standard deviation, of the 3M Petrifilm RAC Plate and the reference method was determined for all environmental surfaces. Cochran and Grubbs outlier tests were performed for the 3M Petrifilm RAC Plate and the reference method results. The statistical analysis between the 3M Petrifilm RAC Plate and the reference method indicated that no outliers were detected and that the methods show no statistically significant differences, with 95% confidence. A summary of the study data, statistical analysis, and 90 and 95% confidence intervals are presented in Table 1. Figures 1–3 display graphs of the log_10_ values of the candidate method and the reference method.

**Table 1.** Matrix study: 3M Petrifilm RAC Plate versus FDA BAM Ch. 3 results

Matrix/Organism	Cont. level^c^	3M Petrifilm RAC Plate results		FDA/BAM Ch. 3 results^a^		DOM^f^	SE^g^	90% CI^b^		95% CI	
		Mean^d^	$s_{r}^{e}$	Mean	s_r_			LCL^h^	UCL^i^	LCL	UCL
Stainless steel/*Listeria innocua* (ATCC 33090)^j^	Non^k^	0	0	0	0	0	0	0	0	0	0
	Low	1.609	0.297	1.531	0.244	0.078	0.078	−0.088	0.244	−0.139	0.294
	Med	2.144	0.156	2.093	0.133	0.051	0.057	−0.072	0.173	−0.109	0.210
	High	3.123	0.107	3.191	0.095	−0.067	0.017	−0.103	−0.032	−0.114	−0.021
Rubber/*Klebsiella aerogenes* (ATCC 35029)	Non	0	0	0	0	0	0	0	0	0	0
	Low	1.823	0.056	1.803	0.101	0.020	0.047	−0.080	0.119	−0.110	0.150
	Med	2.862	0.042	2.819	0.070	0.043	0.041	−0.044	0.130	−0.070	0.156
	High	3.871	0.033	3.801	0.087	0.070	0.029	0.008	0.131	−0.010	0.150
Sealed concrete/*Klebsiella oxytoca* (ATCC 43165)	Non	0	0	0	0	0	0	0	0	0	0
	Low	1.659	0.243	1.788	0.110	−0.129	0.136	−0.419	0.160	−0.506	0.247
	Med	2.136	0.017	2.141	0.042	−0.005	0.016	−0.038	0.028	−0.048	0.038
	High	3.105	0.055	3.124	0.062	−0.019	0.008	−0.036	−0.002	−0.041	0.003

a FDA BAM Ch. 3, Aerobic Plate Count.

b Confidence interval.

c All surfaces are artificially contaminated, 100 cm^2^ test areas.

d Mean of five replicate portions, after logarithmic transformation: log_10_[cfu/g + (0.1)f], where f is the smallest reportable result.

e Repeatability standard deviation.

f Difference of means between the candidate and reference methods.

g Standard error on the mean difference.

h Lower confidence limit for difference of means.

i Upper confidence limit for difference of means.

j American Type Culture Collection, Manassas, VA.

k Noninoculated.

## Discussion

The data support the claim that the 3M Petrifilm RAC Plate shows no statistically significant differences when compared to the BAM Ch. 3 reference method for stainless steel, sealed concrete, and rubber surfaces.

The 3M Petrifilm RAC Plate is robust, quick, and simple to perform, providing enumerable colonies in 22 to 26 h after incubation when compared to the 48 h incubation of the reference method. The small size of the Petrifilm Plate allows for less space to be occupied by plates in the incubators. The addition of visual biochemical and enzymatic indicators makes enumeration of colonies a simple task by presenting colonies in either a blue or red color.

## Conclusions

The data from this study, within their statistical uncertainty, support the product claim that the 3M Petrifilm RAC Plate can be used to enumerate aerobic bacteria on the surface of stainless steel, sealed concrete, and rubber. The results obtained by the analysis of the study demonstrated no statistically significant differences between the number of aerobic bacteria enumerated by the candidate and the reference method for all samples tested.
